# Pulmonary emphysema is a risk factor for radiation pneumonitis in NSCLC patients with squamous cell carcinoma after thoracic radiation therapy

**DOI:** 10.1038/s41598-017-02739-4

**Published:** 2017-06-05

**Authors:** Ziyang Zhou, Xiao Song, Ailu Wu, Hui Liu, Hongyu Wu, Qiongya Wu, Yu Liu, Yefei Li, Yong Cai, Shixiong Liang

**Affiliations:** 1 Department of Radiation Oncology, Shanghai Pulmonary Hospital,Tongji University School of Medicine, Shanghai, 200433 China; 2 Department of Thoracic Surgery, Shanghai Pulmonary Hospital,Tongji University School of Medicine, Shanghai, 200433 China

## Abstract

Pulmonary emphysema (PE) has been demonstrated to have a high prevalence in patients with locally advanced non-small cell lung cancer (NSCLC). A total of 153 patients with locally advanced NSCLC were enrolled in this study to investigate the association between PE and radiation pneumonitis (RP) after definitive thoracic radiation therapy (TRT). The incidence of RP in Grade 2, 3 and 5 were 11.1%, 9.8% and 0.7%, respectively. Univariate analysis revealed that age, PE, forced vital capacity (FVC), arterial partial pressure of oxygen (PO_2_) and mean lung dose (MLD) were significantly associated with the risk of Grade ≥2 or Grade ≥3 RP in patients with squamous cell carcinoma (SCC, *P* < 0.05). Logistic analysis demonstrated that PE was an independent risk factor of RP in SCC (*P* < 0.05). Receiver operating characteristics (ROC) analysis revealed that the combination of age, PE, FVC, PO_2_ and MLD had a higher value to predict RP in SCC (AUC = 0.856 in Grade ≥2 RP and 0.882 in Grade ≥3 RP, respectively). Kaplan-Meier analysis revealed that the more severe the PE, the higher the incidence of RP in SCC. Our results revealed that PE was a high risk factor for locally advanced NSCLC patients followed definitive TRT, especially for SCC patients.

## Introduction

Non-small cell lung cancer (NSCLC) is the most common cancer, and is the most common cause of cancer-related mortality globally. NSCLC accounts for 85% of cases with lung cancer, which includes two major histologic subtypes, squamous cell carcinoma (SCC) and adenocarcinoma (ADE); and these subtypes are the distinction that carries significant clinical and therapeutic implications^1^. Thoracic radiation therapy (TRT) is considered the standard treatment for patients with unresec locally advanced NSCLC. However, normal lung tissue is sensitive to radiation, and radiation pneumonitis (RP) is the most important dose-limiting complication of TRT, which is irreversible and fatal in some cases. The clinical manifestation of RP is usually mild dry cough, fever, or mild dyspnea, and the severe RP could cause extensive pulmonary fibrosis, which may lead to chronic dyspnea and even death. The incidence of moderate and severe RP ranges from 10–20%^2^. RP is the major side effect of TRT, which can impact on the clinical course of patients. Clinical factors and dosimetric factors are two common complications influencing the occurrence of RP. Clinical factors comprise performance status, tumor location, smoking history and concurrent chemotherapy, and dosimetric factors include V5 (volume of total lung receiving ≥5 Gy), V20 (volume of total lung receiving ≥20 Gy), mean lung dose (MLD), irradiated lung volume, total dose and fractionation schedule. These factors have been used to predict the occurrence of RP and have been reported in previous studies^3, 4^.

Chronic obstructive pulmonary disease (COPD) is a common disease characterized by the continuous deterioration of lung function, and has an increased prevalence in China, and present a high prevalence of 40–70% in lung cancer patients^5, 6^. Some recent investigations determined the correlation between **COPD** and RP^7, 8^, which causes oncologists to regard **COPD** as a limitation for TRT in patients of lung cancer due to poor pulmonary function. Pulmonary emphysema (PE) is a crucial subtype of COPD, which is defined pathologically as a group of diseases that demonstrate anatomical alterations in the lung characterized by the enlargement of air spaces distal to the terminal bronchiole and accompanied by destructive changes of the alveolar walls^9^. However, it remains unclear whether the presence of PE is associated with the incidence of RP or severity RP in patients with locally advanced NSCLC. The aim of this study is to evaluate the impact of PE on the incidence of RP in patients with locally advanced NSCLC after definitive TRT.

## Materials and Methods

### Patient demographics

A total of 153 consecutive patents, who were diagnosed as locally advanced NSCLC by histology and radiology and treated with TRT in Shanghai Pulmonary Hospital from January 2014 to January 2015, were enrolled in this study. Recruitment criteria were as follows: (1) patients who had a Karnofsky performance status (KPS) score >70, and could endure a definitive TRT at a total dose of 58–66 Gy (1.8–2.5 Gy/fraction/day); (2) patients who received TRT with concurrent/sequential chemoradiotherapy; (3) patients with thoracic CT images taken prior and during TRT, which are available for evaluation; (4) patients with RP who were followed up for <6 months. The prospective evaluation, which included age, gender, smoking history, histology, stage and PE, were assessed through thoracic CT scans. All patients were staged as IIIA or IIIB based on the classification of tumor/node/metastasis (TNM) stages (International Union Against Cancer TNM classification, 7th edition)^10^, and were thereby considered inoperable. Patients with severe PE or poor lung function were considered not suitable for concurrent chemoradiotherapy, and the older patients were reluctant to choose this treatment mode due to poor endurance. All methods mentioned in the protocol were carried out in accordance with institutional guidelines and were approved by the Ethical Review Committee of Shanghai Pulmonary Hospital, Tongji University. Informed consent was obtained from all patients.

### Radiotherapy

All patients received a planning CT scan and were immobilized in the supine position with their arms raised in a customized alpha-cradle mold. The simulation CT images were taken in 5-mm increments over the region of interest. Gross tumor volume (GTV) consisted of all known sites of the disease and considered large if it was ≥100 cc. Treatment planning was performed with the ADAC pinnacle treatment planning system. Treatments were delivered with 6 MV photons by 3D-conformal radiation therapy (3D-CRT) or intensity modulated radiation therapy (IMRT) on a linear accelerator (Clinac 21EX, Varian Medical Systems, USA) or a Siemens Artiste (Oncology Care Systems, Siemens Medical Solutions, Concord, CA, USA) digital linear accelerator with a multi-leaf collimator. A planning target volume (PTV) was established to add margins for GTV and for beam position error, which was defined by the radiation oncologist. The planning goal was to deliver the prescription dose to at least 95% of the PTV, while meeting normal tissue constraints. The median total dose was 60 Gy (range: 58–66 Gy), and the fraction dose was 1.8–2.5 Gy (median 2.0 Gy). Patients who underwent stereotactic body radiation therapy (SBRT) were excluded from this study. Dose parameters V5, V10, V15, V20 and V25 were obtained as the percentages of the pulmonary volume irradiated to >5, >10, >15, >20 and >25 Gy, respectively. At the same time, mean lung dose (MLD) was calculated. The complete dose-volume histogram (DVH) data were available for all patients.

### Chemotherapy

All patients received chemotherapy. Among these patients, 22 patients were concurrently given chemotherapy, and 131 patients were sequentially given chemotherapy. Among these 131 patients, 123 patients received chemotherapy first and eight patients received TRT first. Although there was no specific chemotherapy protocol, the former usually consisted of GP (gemcitabine and cisplatin; 69 patients, 45.1%), NP (vinorelbine and cisplatin; 20 patients, 13.1%), or TP (toxal and cisplatin; 15 patients, 9.8%). Two courses of consolidation chemotherapy were administered in 69 patients (45.1%).

### Evaluation of PE and RP in CT images

**Figure 1:**
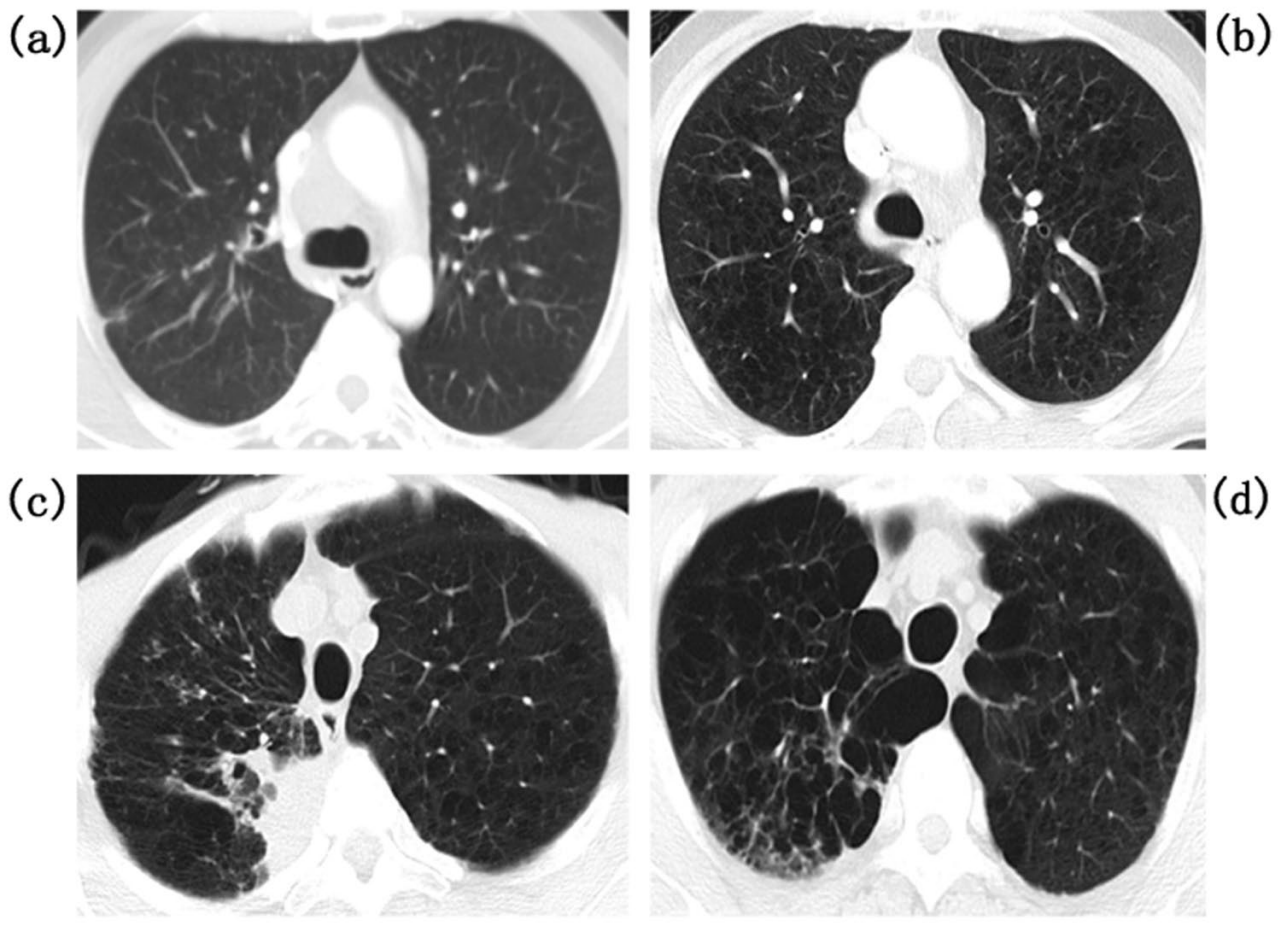
Pulmonary emphysema (PE) in computed tomographic (CT) images: (**a**) Grade 0, no low-attenuation areas (LAAs); (**b**) Grade 1, sparse and scattered small LAAs up to 5 mm in diameter; (**c**) Grade 2, adjacent LAAs up to 10 mm in diameter; (**d**) Grade 3, adjacent or indistinguishable LAAs > 10 mm in diameter.

**Figure 2:**
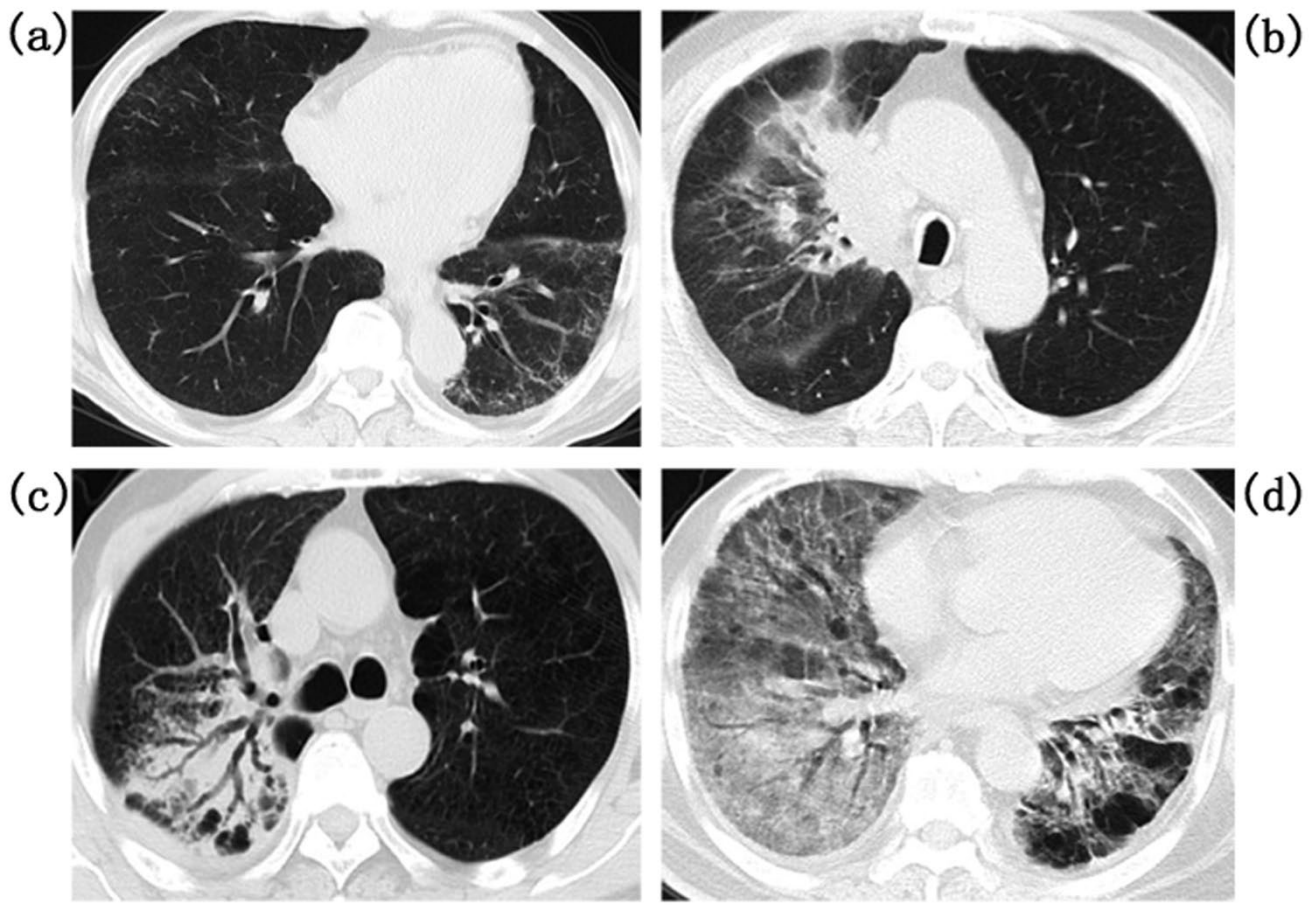
Radiation pneumonitis (RP) in computed tomographic (CT) images: (**a**) Grade 1, (**b**) Grade 2, (**c**) Grade 3, and (**d**) Grades 4–5.

PE was assessed according to the low-attenuation areas (LAAs) on CT scans taken prior to treatment, and was classified into five grades based on Satoh K’s criteria^11^: no LAAs was classified as Grade 0, sparse and scattered small LAAs up to 5 mm in diameter was classified as Grade 1, adjacent LAAs up to 10 mm in diameter was classified as Grade 2, adjacent or indistinguishable LAAs >10 mm in diameter was classified as Grade 3, and almost no present normal lung parenchyma was classified as Grade 4. The PE grades according to our CT scans are presented in Fig. 1. No patient suffered Grade 4 PE in the present study. According to the Common Terminology Criteria for Adverse Events 4.0, RP was diagnosed through clinical symptoms and the radiographic changes on the CT scans of patients. These patients were graded as follows: Grade 0, no symptom or radiographic change; Grade 1, asymptomatic and radiographic changes only; Grade 2, symptomatic but did not interfering with the daily life of the patient; Grade 3, symptomatic, interfered with the daily life of the patient, and O_2_ was needed; Grade 4, life-threatening and ventilator support indicated; Grade 5, death. RP was graded by several experienced radiation oncologists at our institution to ensure the veracity of the grading. The RP grades based on the CT scans are shown in Fig. 2.

### Follow-up

A CT scan prior to treatment was necessary for all patients, which was basically planed at the first 1–2 months after TRT. Patients were follow-up weekly up to the end of TRT and every 1–3 months thereafter, in order to evaluate the curative effect and determine adverse events. The follow-up period lasted for more than one year, as the patient’s physical status remained stable. The endpoint was the incidence of Grade ≥2 RP.

### Statistical analysis

The association between RP and clinical factors were analyzed using Person Chi-square test or Continuity Correction test. The relationships between PE score and RP Grade were assessed using Spearman’s correlation test. Receiver operating characteristic (ROC) curves and Kaplan-Meier methods were carried out to analyze possible predictors and the cumulative incidence rates of Grade ≥2 and Grade ≥3 RP, respectively. Multivariate logistic regression analyses were performed to evaluate the data for the association between clinical factors and DVH parameters with RP. Statistical analyses were performed using SPSS software 13.0. A *P*-value < 0.05 was considered significant.

## Results

### Incidence of radiation pneumonitis and pulmonary emphysema

140 male and 13 female patients were enrolled in this study. The characteristics of these patients are summarized in Table 1. The median follow-up was 10 months for all patients. The incidence of Grade ≥2 RP was 21.6% (33 patients), including 1 patient (0.7%) with fatal RP (Grade 5) and 15 patients (9.8%) with Grade 3 RP. No patient suffered from Grade 4 RP. The occurrence of RP ranged from 1 to 11 months (4.1 ± 2.8 months) after the start of TRT. PE occurred in 68 patients, including 45 patients (29.4%) in Grade 1, 22 patients (14.4%) in Grade 2, and 1 patient (0.7%) in Grade 3.

**Table 1:** Demographics of patients.

Characteristic (*n* = 153)		*n*
Gender	Male	140 (91.5%)
	Female	13 (8.5%)
Age(years)	Range	40–85
	Median	63
Pathology	Adenocarcinoma	28 (18.3%)
	Squamous cell carcinoma	93 (60.8%)
	Others^a^	32 (20.9%)
Stage	IIIA	55 (35.9%)
	IIIB	98 (64.1%)
Smoking history	Yes	94 (61.4%)
	No	59 (38.6)
Tumor location	Upper lobe	87 (56.9%)
	Middle lobe	29 (18.9%)
	Lower lobe	37 (24.2%)
Chemotherapy	Concurrent	22 (14.4%)
	Sequence 1^b^	123 (80.4%)
	Sequence 2^c^	8 (5.2%)
RP Grade^d^	0–1	120 (78.4%)
	2	17 (11.1%)
	3	15 (9.8%)
	5	1 (0.7%)

^a^Other types of pathology included adenosquamous carcinoma and some NSCLC with a non-clear diagnosis. ^b^Sequence 1, patients who received chemotherapy first. ^c^Sequence 2, patients who received radiation therapy first. ^d^RP, radiation pneumonitis.

### Risk factors associated with radiation pneumonitis

In order to investigate the correlative predictors of RP, the univariate analysis was performed to evaluate clinical and dosimetric factors with the incidence of RP. Results revealed that patients with higher PE Grade and forced vital capacity (FVC) <2.8 L had a higher risk to the development of Grade ≥2RP (*P* = 0.012 and *P* = 0.022, respectively). Gender, primary tumor location, pathology, smoking history, forced expiratory volume in 1 second (FEV1)/FVC%, arterial partial pressure of oxygen (PO_2_), chemotherapy modality (sequential or concurrent), as well as dosimetric parameters including V5, V20 and MLD, were not related to the risk of developing Grade ≥2 RP (all *P* > 0.05, Table 2). In order to perform further investigation, these patients were divided into two groups for a subgroup analysis based on the different pathologies. Results revealed that there was no risk factor for patients with non-squamous cell carcinoma patients to suffer from Grade ≥2 RP, but for patients with SCC, age (*P* = 0.014), PE (*P* < 0.001), FVC (*P* = 0.007) and PO_2_ (*P* = 0.02) presented significant association with the development of Grade ≥2 RP.

**Table 2:** Univariate analysis of possible risk factors associated with the development of Grade ≥2 RP.

Risk factors		Non-small cell lung cancer (n = 153)			Non-squamous cell carcinoma (n = 60)			Squamous cell carcinoma (n = 93)		
		RP<G2 (*n*%)	RP ≥G2 (*n*%)	*P*-value	RP<G2 (*n*%)	RP ≥G2 (*n*%)	*P*-value	RP<G2 (*n*%)	RP ≥G2 (*n*%)	*P*-value
Gender	Male	110	30	>0.999	38	11	>0.999	72	19	>0.999
	Female	10	3		8	3		2	0	
Age		62.19 ± 8.89	65.70 ± 9.58	0.050	63.26 ± 9.31	63.71 ± 10.29	0.613	61.53 ± 8.62	67.16 ± 9.02	**0.014**
Tumor location	Upper	67	20	0.656	23	7	0.703	44	13	0.736
	Middle	22	7		9	4		13	3	
	Lower	31	6		14	3		17	3	
Smoking history	Yes	75	19	0.607	25	6	0.451	50	13	0.943
	No	45	14		21	8		24	6	
chemotherapy	Concurrent	20	2	0.248	9	0	0.059	11	2	0.424
	Sequence 1^a^	94	29		34	12		60	17	
	Sequence 2^b^	6	2		3	2		3	0	
PE Grade	0	73	12	**0.012**	29	8	0.162	44	4	<**0.001**
	1	34	11		12	6		22	5	
	≥2	13	10		5	0		8	10	
FEV1/FVC%	≥70	56	15	0.902	22	8	0.542	34	7	0.476
	<70	64	18		24	6		40	12	
FVC	≥2.8	67	11	**0.022**	22	6	0.744	45	5	**0.007**
	<2.8	53	22		24	8		29	14	
PO_2_		84.13 ± 11.85	81.42 ± 9.07	0.225	81.43 ± 13.40	84.00 ± 7.92	0.085	85.81 ± 10.54	79.53 ± 9.58	**0.020**
PCO_2_		37.49 ± 4.09	36.93 ± 4.51	0.497	37.33 ± 4.03	36.87 ± 4.67	0.813	37.59 ± 4.15	36.98 ± 4.52	0.573
V5		49.14 ± 7.64	50.52 ± 7.51	0.360	49.28 ± 7.44	50.36 ± 8.46	0.680	49.05 ± 7.81	50.63 ± 6.96	0.425
V20		22.16 ± 3.96	23.55 ± 3.96	0.077	21.93 ± 3.99	23.50 ± 4.36	0.565	22.30 ± 3.97	23.58 ± 3.76	0.208
MLD		11.88 ± 2.61	12.69 ± 2.67	0.118	11.96 ± 2.60	12.47 ± 3.21	0.210	11.83 ± 2.63	12.85 ± 2.27	0.125

RP: radiation pneumonitis; G2, grade 2; G3, grade 3; PE, pulmonary emphysema; FEV1, forced expiratory volume in one second; FVC, forced vital capacity; PO_2_, arterial partial pressure of oxygen; PCO_2_, arterial partial pressure of carbon dioxide; V5, V20, the percentages of pulmonary volume irradiation exceeding 5 Gy, 20 Gy; MLD, mean lung dose. ^a^Sequence 1, patients who received chemotherapy first. ^b^Sequence 2, patients who received radiation therapy first. Significant differences are indicated in bold.

The univariate analysis results for risk factors associated with Grade ≥3 RP are presented in Table 3. The results showed that higher PE Grade (*P* = 0.006) and older age (*P* = 0.009) were significantly associated with the development of Grade ≥3 RP in NSCLC. Furthermore, the stratified analysis revealed that V5 was the only factor related to Grade ≥3 RP (*P* = 0.01) in non-squamous cell carcinoma patients, while in patients with SCC, older age (*P* = 0.032), higher PE grade (*P* = 0.007) and higher mean lung dose (MLD, *P* = 0.037) presented significant results in Grade ≥3 RP. The results of unconditional logistic analysis showed that PE was the major factor with the association of Grade ≥2 RP (OR = 1.985, *P* = 0.01) and Grade ≥3 RP (OR = 2.275, *P* = 0.02) in NSCLC. Subgroup analysis showed that PE was the independent factor with the development of Grade ≥2 RP (OR = 3.304, *P* = 0.002) and Grade ≥3 RP (OR = 2.718, *P* = 0.031) in patients with SCC (Table 4).

**Table 3:** Univariate analysis of possible risk factors associated with the development of Grade ≥3 RP.

Risk factors		Non-small cell lung cancer (n = 153)			Non-squamous cell carcinoma (n = 60)			Squamous cell carcinoma (n = 93)		
		RP <G3 (*n*%)	RP ≥G3 (*n*%)	*P*-value	RP <G3 (*n*%)	RP ≥G3 (*n*%)	*P*-value	RP <G3 (*n*%)	RP ≥G3 (*n*%)	*P*-value
Gender	Male	125	15	>0.999	44	5	>0.999	81	10	>0.999
	Female	12	1		10	1		2	0	
Age		62.29 ± 8.90	68.56 ± 9.46	**0.009**	62.76 ± 9.52	68.83 ± 7.36	0.307	61.99 ± 8.51	68.40 ± 10.91	**0.032**
Tumor location	Upper	77	10	0.847	27	3	0.692	50	7	0.765
	Middle	26	3		11	2		15	1	
	Lower	34	3		16	1		18	2	
Smoking history	Yes	84	10	0.927	28	3	>0.999	56	7	>0.999
	No	53	6		26	3		27	3	
Chemotherapy	Concurrent	21	1	0.559	9	0	0.304	12	1	0.641
	Sequence 1^a^	109	14		41	5		68	9	
	Sequence 2^b^	7	1		4	1		3	0	
PE Grade	0	82	3	**0.006**	35	2	0.112	47	1	**0.007**
	1	37	8		14	4		23	4	
	≥2	18	5		5	0		13	5	
FEV1/FVC %	≥70	65	6	0.450	27	3	>0.999	38	3	0.540
	<70	72	10		27	3		45	7	
FVC	≥2.8	72	6	0.254	25	3	>0.999	47	3	0.208
	<2.8	65	10		29	3		36	7	
PO_2_		83.97 ± 11.71	79.94 ± 6.72	0.179	82.19 ± 12.86	80.67 ± 6.19	0.307	85.13 ± 10.81	79.50 ± 7.31	0.113
PCO_2_		37.54 ± 4.27	35.98 ± 3.05	0.158	37.38 ± 4.26	35.82 ± 2.89	0.103	37.64 ± 4.30	36.07 ± 3.30	0.269
V5		49.25 ± 7.75	51.06 ± 6.17	0.368	49.37 ± 8.02	51.00 ± 1.10	**0.010**	49.17 ± 7.62	51.10 ± 7.92	0.453
V20		22.27 ± 3.98	24.06 ± 3.84	0.089	22.11 ± 4.12	24.00 ± 3.74	0.562	22.37 ± 3.91	24.10 ± 4.09	0.192
MLD		11.93 ± 2.64	13.09 ± 2.39	0.098	12.07 ± 2.78	12.17 ± 2.49	0.882	11.85 ± 2.56	13.64 ± 2.27	**0.037**

RP: radiation pneumonitis; G2, grade 2; G3, grade 3; PE, pulmonary emphysema; FEV1, forced expiratory volume in one second; FVC, forced vital capacity; PO_2_, arterial partial pressure of oxygen; PCO_2_, arterial partial pressure of carbon dioxide; V5, V20, the percentages of pulmonary volume irradiation exceeding 5 Gy, 20 Gy; MLD, mean lung dose. ^a^Sequence 1, patients who received chemotherapy first. ^b^Sequence 2, patients who received radiation therapy first. Significant differences are indicated in bold.

**Table 4:** Unconditional logistic analysis of possible risk factors associated with the development of RP.

Risk factors	NSCLC patients with RP ≥G2		NSCLC patients with RP ≥G3		SCC patients with RP ≥G2		SCC patients with RP ≥G3	
	OR (95% CI)	*P*-value	OR (95% CI)	*P*-value	OR (95% CI)	*P*-value	OR (95% CI)	*P*-value
Age	1.028 (0.980–1.079)	0.259	1.078 (1.004–1.157)	**0.037**	1.043 (0.972–1.120)	0.240	1.058 (0.966–1.159)	0.221
PE Grade	1.985 (1.174–3.354)	**0.010** ^a^	2.275 (1.141–4.533)	**0.020** ^a^	3.304 (1.555–7.023)	**0.002** ^a^	2.718 (1.096–6.740)	**0.031** ^**a**^
FVC	2.403 (0.960–6.017)	0.061	1.131 (0.321–3.980)	0.848	3.681 (0.974–13.914)	0.055	2.049 (0.381–11.020)	0.403
PO_2_	1.002 (0.964–1.041)	0.928	0.986 (0.936–1.039)	0.597	0.951 (0.892–1.013)	0.119	0.958 (0.884–1.038)	0.292
MLD	1.129 (0.962–1.326)	0.137	1.192 (0.951–1.493)	0.128	1.170 (0.925–1.481)	0.191	1.336 (0.967–1.844)	0.079

^a^ *P* for the trend of grade 0, Grade 1 and Grade ≥2; RP, radiation pneumonitis; OR, odds ratio; NSCLC, non-small cell lung cancer; SCC, squamous cell carcinoma; G2, Grade 2; G3, Grade 3; PE, pulmonary emphysema; FVC, forced vital capacity; PO_2_, arterial partial pressure of oxygen; MLD, mean lung dose. Significant differences are indicated in bold.

### Receiver operating characteristics (ROC) analysis

**Figure 3:**
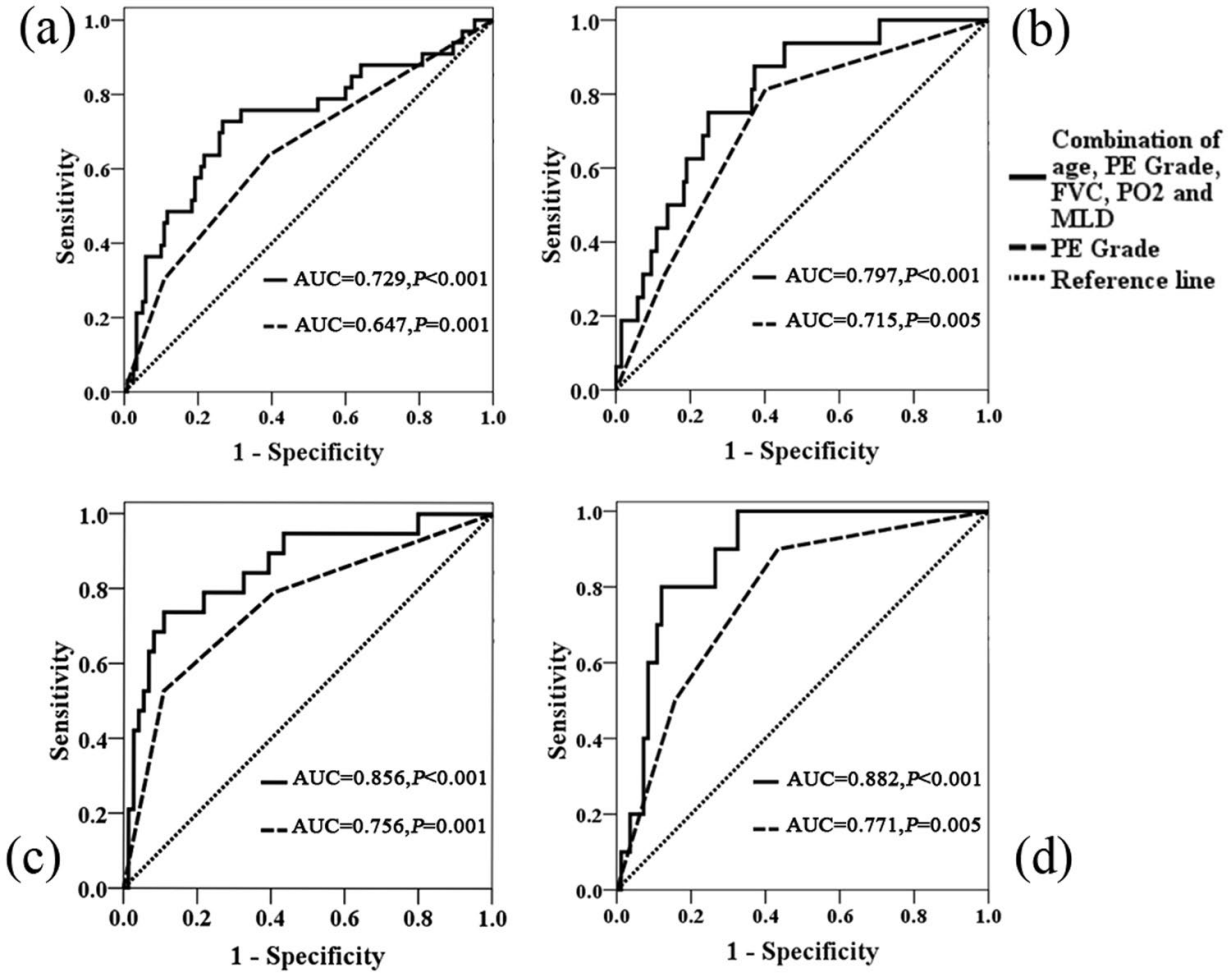
Receiving operator characteristic (ROC) curve based on the sensitivity and specificity of pulmonary emphysema (PE) alone, and the combination of age, PE, forced vital capacity (FVC), arterial partial pressure of oxygen (PO_2_) and mean lung dose (MLD). (**a**) ROC curve of Grade ≥2 RP in non-small cell lung cancer (NSCLC); (**b**) ROC curve of Grade ≥3 RP in NSCLC; (**c**) ROC curve of Grade ≥2 RP in squamous cell carcinoma (SCC); (**d**) ROC curve of Grade ≥3 RP in SCC.

**Figure 4:**
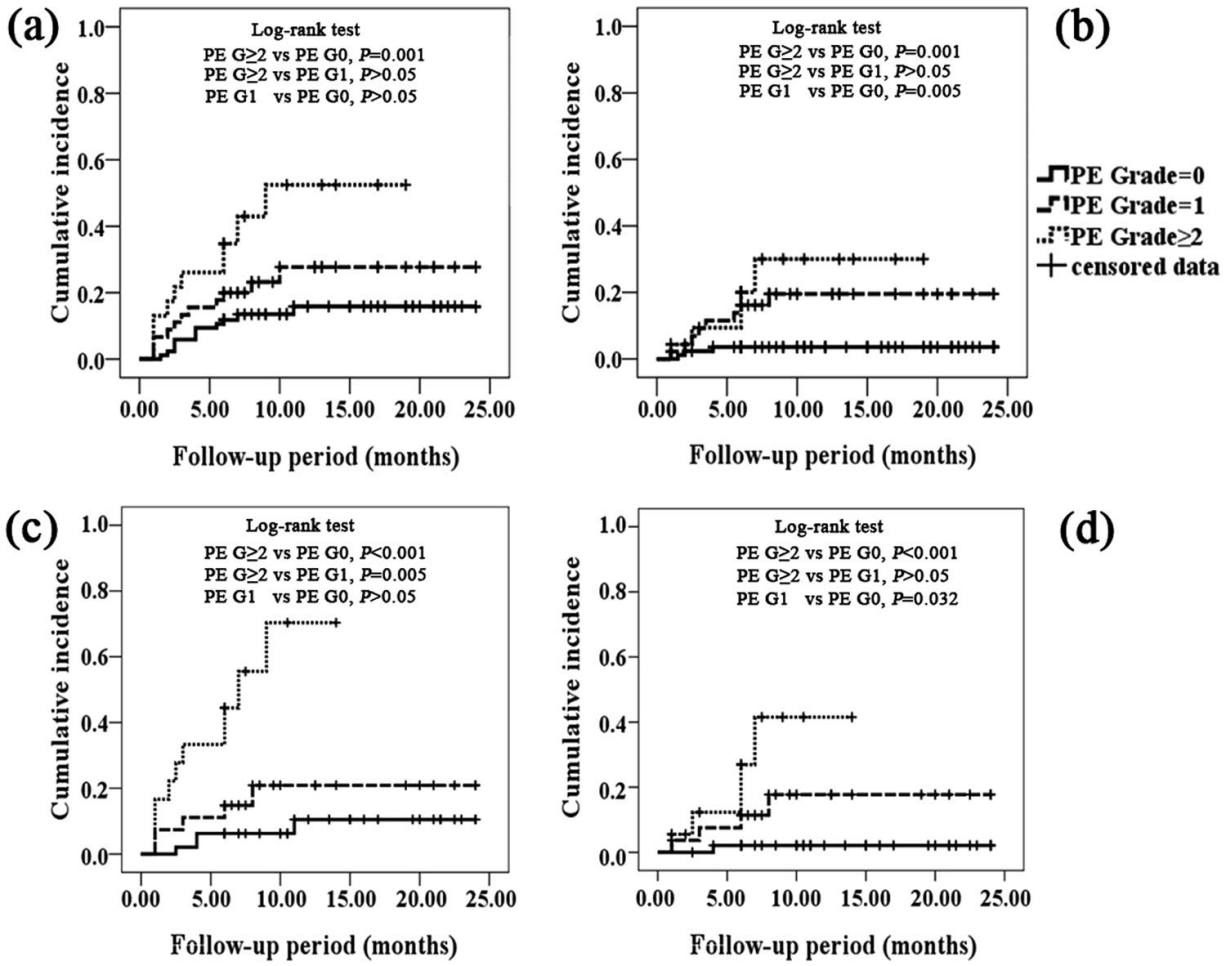
The cumulative incidence of radiation pneumonitis (RP) stratified by pulmonary emphysema (PE). (**a**) The cumulative incidence of Grade ≥2 RP in non-small cell lung cancer (NSCLC); (**b**) the cumulative incidence of Grade ≥3 RP in NSCLC; (**c**) the cumulative incidence of Grade ≥2 RP in squamous cell carcinoma (SCC); (**b**) the cumulative incidence of Grade ≥3 RP in SCC.

The multivariate analysis revealed that PE was the only independent risk factor for the incidence of RP in patients with SCC, and the relationship between PE and RP was analyzed using Spearman’s analysis. Results revealed that PE Grade was positively correlated with RP Grade (rho = 0.249, *P* = 0.002). In addition, the ROC curve revealed that the area under the curve (AUC) of PE in NSCLC was 0.647 (*P* = 0.01) in Grade ≥2 RP and 0.715 (*P* < 0.001) in Grade ≥3 RP; and with the combination of the five factors (PE, age, FVC, PO_2_ and MLD), the AUC was up to 0.729 (*P* < 0.001) in Grade ≥2 RP and 0.797 (*P* < 0.001) in Grade ≥3 RP (*P* < 0.001). However, in patients with SCC, the AUC of PE was 0.756 (*P* = 0.001) in Grade ≥2 RP and 0.771 (*P* = 0.005) in Grade ≥3 RP; and with the combination of the five factors (PE, age, FVC, PO_2_ and MLD), the AUC was up to 0.856 (*P* < 0.001) in Grade ≥2 RP and 0.882 (*P* < 0.001) in Grade ≥3 RP (*P* < 0.001) (Fig. 3). The cumulative incidence of Grade ≥3 RP and Grade ≥2 RP in different PE Grades was calculated using Kaplan-Meier methods (Fig. 4). The events of Grade ≥2 RP in NSCLC patients with PE Grade 0, PE Grade 1 and PE Grade ≥2 were 12 (14.1%), 11 (24.4%) and 10 (43.5%), respectively, while the events of Grade ≥3 RP in NSCLC patients with PE Grade 0, PE Grade 1 and PE Grade ≥2 were 3 (3.5%), 8 (17.8%) and 5 (21.7%), respectively. The incidence of Grade ≥2 RP in SCC patients with PE Grade 0, PE Grade 1 and PE Grade ≥2 were 4 (8.3%), 5 (18.5%) and 10 (55.6%), respectively; and the incidence of Grade ≥3 RP in SCC patients with PE Grade 0, PE Grade 1 and PE Grade ≥2 were 1 (2.1%), 4 (14.8%) and 5 (27.8%), respectively.

## Discussion

RP remains a major problem that may result in considerable morbidity and limit the dose of radiation treatment in NSCLC patients^12^. Grade ≥2 RP was considered as a symptomatic RP that requires intervention treatment^13^, and Grade ≥3 RP is regarded as severe RP that could lead to poor prognosis^14^. As progress has been made in understanding the underlying mechanism of RP, more work is needed to develop a safe and effective means of preventing and treating this condition. This prospective study aimed to evaluate the incidence rate and risk factors of RP after TRT for lung cancer in a large single institution series. Dosimetric factors such as V20 and MLD are considered to be highly correlated with the incidence of RP^12, 15^. Some investigations have implied that V20 ≥ 25% or MLD ≥14 Gy would be a risk factor to increase the incidence of severe RP^14, 16^. In this study, although the TRT dose was reduced to a safety range: the mean value of V20 and MLD was <25% and 14 Gy, the occurrence of RP was still similar to other researches^2, 14^. This suggests that other clinical factors or systemic agents are involved in the occurrence of RP. Our investigation revealed that factors including age, PE, PO_2_, FVC and MLD were significantly associated with the development of RP, and the presence of PE was the only independent risk factor for the development of symptomatic or severe RP. In the stratified analysis, risk factors such as age, PE, FVC and PO_2_ were more significant in patients with SCC. However, no significant results were detected in non-squamous cell carcinoma patients. Furthermore, the results of the present study revealed that the severity of PE was correlated to the higher cumulative incidence of symptomatic or severe RP in locally advanced NSCLC patients with SCC after TRT. Moreover, the combination of age, PE, FVC, PO_2_ and MLD had a higher value to predict the occurrence of RP.

Although the relationship between RP and COPD including PE has been investigated in recent years, it still remains controversial. Some researchers have pointed out that patients with COPD or PE have a higher risk of suffering from RP^8, 15^, while other researchers reported that patients with severe COPD or PE had lower risk of suffering from RP followed by SBRT^16, 17^. In addition, other researchers have shown that PE was irrelevant to the incidence of RP after TRT^13, 14^. Our results revealed that PE was a risk factor related to RP, but this association only existed in NSCLC patients with SCC. This is a new finding for radiation oncologists, because few researches have focused on the risk of RP between different types of pathology in NSCLC. The main reasons considered are as follows: First, the majority of SCC have been considered to occur centrally and grow largely. Hence, the GTV of patients with SCC are usually larger than those patients in other types of pathology, and the irradiated area in residual lung parenchyma were also larger. Second, the central SCC may obstruct the airway and lead to lower pulmonary function, which would cause an imbalance between oxidants and antioxidants and create a higher level of reactive oxygen species (ROS)^18^. Those factors could positively enhance lung injury. Finally, local and systemic chronic inflammation, which was the main characteristic of COPD and PE, might cause chronic mitogenesis and increase the likelihood of DNA damage^19^. This mechanism was considered to be a trigger of RP^20^.

The association between age and RP was also investigated in recent studies. Tsujino *et al*.^14^ reported that an age of ≥68 was a risk factor in their model for predicting severe RP in a retrospectively study of 122 patients, who had a median age of 63 years old. A literature-based meta-analysis pooled 11 studies for age comparison, and concluded that older patients were more likely to increase RP risk^21^. Some other studies revealed that age was not a significant risk factor associated with RP^13, 16^. It is noteworthy that these studies commonly used the median age as the dichotomizing value, while the practice of dichotomizing continuous covariates was considered unnecessary for statistical analysis^22^. Additionally, poor pulmonary function tests did not increase the risk of RP, as been reported in some investigations^14, 16^. They suggested that TRT could also be a good treatment option for patients with NSCLC combined with COPD or PE. However, in a prospective study, Jeba *et al*.^23^ revealed that there was a universal fall in all pulmonary function parameters except for Diffusing Capacity for Carbon Monoxide (DLCO) in patients with RP. Hence, the main consideration on the adverse reaction of TRT in patients with COPD was not RP, but the worsening of lung function. Furthermore, no study has reported on whether age or pulmonary function factors could be a good predictor for RP in patients with SCC. Our data revealed an insignificant value in the correlation of RP and age or pulmonary function after the multivariate analysis. However, the combination of the five factors (age, PE, FVC, PO_2_ and MLD) provided a higher accuracy to predict RP than PE only. Consequently, careful observation and management of RP should be provided after TRT in such patients with older age, higher grades of PE and severe pulmonary function.

There were some limitations in this study. Concurrent chemoradiotherapy is usually the standard treatment for patients with locally advanced NSCLC^24^. However, cases that underwent concurrent chemoradiotherapy were less than the other studies in our data. After the statistics analysis, we found that there was no difference in RP rate, regardless of whether patients accepted concurrent chemoradiotherapy or not; and this coincided with the results of other investigations^14, 16^. Nevertheless, concurrent chemoradiotherapy should be encouraged for patients with a good condition to endure the adverse reaction, in order to improve the local control of NSCLC. Another limitation is that the diagnosis of PE was evaluated through CT images, which lacks prospective data or physiological data to demonstrate the correlation of PE and RP. In addition, the number of patients with Grade ≥3 PE was not enough to draw a definitive conclusion, which would reduce the statistical power of this study. Therefore, further prospective studies will be needed to assess our results.

In summary, our study focused on the investigation of risk factors for the occurrence of RP in locally advanced NSCLC patients. It was revealed that age, PE, FVC, PO_2_ and MLD were significantly associated with the incidence of RP. Further analysis revealed that PE was an independent risk factor for RP. The combination of age, PE, FVC, PO_2_ and MLD had a higher value to predict the occurrence of RP in SCC, than in NSCLC. PE was a high risk factor for locally advanced NSCLC patients after definitive TRT, especially for SCC patients.

## References

[1] Siegel R, Naishadham D, Jemal A (2013). Cancer statistics, 2013. CA: a cancer journal for clinicians.

[2] Mehta V (2005). Radiation pneumonitis and pulmonary fibrosis in non-small-cell lung cancer: pulmonary function, prediction, and prevention. International journal of radiation oncology, biology, physics.

[3] Seppenwoolde Y, Lebesque JV (2001). Partial irradiation of the lung. Seminars in radiation oncology.

[4] Rodrigues G, Lock M, D’Souza D, Yu E, Van Dyk J (2004). Prediction of radiation pneumonitis by dose - volume histogram parameters in lung cancer–a systematic review. Radiotherapy and oncology: journal of the European Society for Therapeutic Radiology and Oncology.

[5] Loganathan RS, Stover DE, Shi W, Venkatraman E (2006). Prevalence of COPD in women compared to men around the time of diagnosis of primary lung cancer. Chest.

[6] Young RP (2009). COPD prevalence is increased in lung cancer, independent of age, sex and smoking history. The European respiratory journal.

[7] Kocak Z (2005). Challenges in defining radiation pneumonitis in patients with lung cancer. International journal of radiation oncology, biology, physics.

[8] Rancati T, Ceresoli GL, Gagliardi G, Schipani S, Cattaneo GM (2003). Factors predicting radiation pneumonitis in lung cancer patients: a retrospective study. Radiotherapy and oncology: journal of the European Society for Therapeutic Radiology and Oncology.

[9] Standards for the diagnosis and care of patients with chronic obstructive pulmonary disease (COPD) and asthma. (1987). This official statement of the American Thoracic Society was adopted by the ATS Board of Directors, November 1986. The American review of respiratory disease.

[10] Detterbeck FC, Boffa DJ, Tanoue LT (2009). The new lung cancer staging system. Chest.

[11] Satoh K (2001). CT assessment of subtypes of pulmonary emphysema in smokers. Chest.

[12] Kocak Z (2007). Prospective assessment of dosimetric/physiologic-based models for predicting radiation pneumonitis. International journal of radiation oncology, biology, physics.

[13] Yamaguchi S (2015). Radiotherapy for thoracic tumors: association between subclinical interstitial lung disease and fatal radiation pneumonitis. International journal of clinical oncology.

[14] Tsujino K (2014). Combined analysis of V20, VS5, pulmonary fibrosis score on baseline computed tomography, and patient age improves prediction of severe radiation pneumonitis after concurrent chemoradiotherapy for locally advanced non-small-cell lung cancer. Journal of thoracic oncology: official publication of the International Association for the Study of Lung Cancer.

[15] Kharofa J, Gore E (2013). Symptomatic radiation pneumonitis in elderly patients receiving thoracic irradiation. Clinical lung cancer.

[16] Kimura T (2012). Radiation pneumonitis in patients with lung and mediastinal tumours: a retrospective study of risk factors focused on pulmonary emphysema. The British journal of radiology.

[17] Ishijima M (2015). Patients with severe emphysema have a low risk of radiation pneumonitis following stereotactic body radiotherapy. The British journal of radiology.

[18] Jaroenpool, J., Pattanapanyasat, K., Noonin, N. & Prachongsai, I. Increased Reactive Oxygen Species (ROS) as Aberrant Neutrophil Function among Healthy Smokers with High Cigarette Smoking Profile and Chronic Obstructive Pulmonary Disease Patients. *Asian Pacific journal of allergy and immunology / launched by the Allergy and Immunology Society of Thailand* (2016). 10.12932/AP0724 27001650

[19] Lee G, Walser TC, Dubinett SM (2009). Chronic inflammation, chronic obstructive pulmonary disease, and lung cancer. Current opinion in pulmonary medicine.

[20] Xue J (2013). Gene-modified mesenchymal stem cells protect against radiation-induced lung injury. Molecular therapy: the journal of the American Society of Gene Therapy.

[21] Vogelius IR, Bentzen SM (2012). A literature-based meta-analysis of clinical risk factors for development of radiation induced pneumonitis. Acta oncologica (Stockholm, Sweden).

[22] Naggara O (2011). Analysis by categorizing or dichotomizing continuous variables is inadvisable: an example from the natural history of unruptured aneurysms. AJNR. American journal of neuroradiology.

[23] Jeba J (2015). Radiation Pneumonitis After Conventional Radiotherapy For Breast Cancer: A Prospective Study. Journal of clinical and diagnostic research: JCDR.

[24] Glatzer M, Elicin O, Ramella S, Nestle U, Putora PM (2016). Radio(chemo)therapy in locally advanced nonsmall cell lung cancer. European respiratory review: an official journal of the European Respiratory Society.

